# A dataset mapping the potential biophysical effects of vegetation cover change

**DOI:** 10.1038/sdata.2018.14

**Published:** 2018-02-20

**Authors:** Gregory Duveiller, Josh Hooker, Alessandro Cescatti

**Affiliations:** 1 European Commission Joint Research Centre, Directorate D-Sustainable Resources-Bio-Economy Unit, I - 21027 Ispra (VA), Italy

**Keywords:** Climate-change mitigation, Ecosystem ecology, Environmental chemistry

## Abstract

Changing the vegetation cover of the Earth has impacts on the biophysical properties of the surface and ultimately on the local climate. Depending on the specific type of vegetation change and on the background climate, the resulting competing biophysical processes can have a net warming or cooling effect, which can further vary both spatially and seasonally. Due to uncertain climate impacts and the lack of robust observations, biophysical effects are not yet considered in land-based climate policies. Here we present a dataset based on satellite remote sensing observations that provides the potential changes i) of the full surface energy balance, ii) at global scale, and iii) for multiple vegetation transitions, as would now be required for the comprehensive evaluation of land based mitigation plans. We anticipate that this dataset will provide valuable information to benchmark Earth system models, to assess future scenarios of land cover change and to develop the monitoring, reporting and verification guidelines required for the implementation of mitigation plans that account for biophysical land processes.

## Background & Summary

Changes in vegetation cover influence the climate through both biogeochemical and biophysical mechanisms^1–3^. The biogeochemical effect of land processes such as deforestation (typically a net emission of CO_2_ into the atmosphere), has global consequences and is at the centre of climate agreements. The biophysical effects are more local in nature and often result from more complex and bidirectional land-climate interactions^4^. For example, converting forests to grasslands typically causes a rapid increase in albedo^5^, but also a decrease in evapotranspiration (because grasses typically have shallower roots and thus access less water). This land conversion may ultimately lead to cooling or warming, depending on which of the two processes dominates^6–8^. In cold climates the albedo effect is amplified by snow cover as trees are more effective than grasses in masking out the radiative cooling effect from snow on the ground, while in water limited regions variations in evapotranspiration become more relevant. These processes clearly show how the biophysical effects of land cover change can vary in sign and magnitude depending on the background climate^9^ and must therefore be quantified at local levels.

To date policies tackling climate mitigation through land management, such as REDD+ (Reducing Emissions from Deforestation and Forest Degradation) focus only on biogeochemical mechanisms and neglect their biophysical consequences, partly because there is a lack of adequate observational data describing the potential biophysical effects of land cover change. Several strategies have been used to quantify these biophysical effects following perturbations in vegetation cover. Assessments based on ground observations, such as those from flux sites and meteorological stations^10–13^, provide a valuable insight but typically have insufficient spatial coverage to derive conclusions at the global scale. Earth system models have also been used to simulate complex land-climate biophysical interactions^14–17^, but the capacity of such models to represent accurately these biophysical properties and, in particular, the partitioning of available energy into latent and sensible heat fluxes, is still uncertain^14,18^. A third way that has gained increased traction lies in exploiting the capacity of satellite remote sensing observations to derive different diagnostics at different scales^5,7,8,19,20^. Although these studies vary in complexity and scope, none have resulted in an explicit spatial dataset describing potential changes in i) the full energy balance, ii) at global scale, and iii) for multiple vegetation transitions, as would now be required for the comprehensive evaluation of land based mitigation plans.

**Figure 1:**
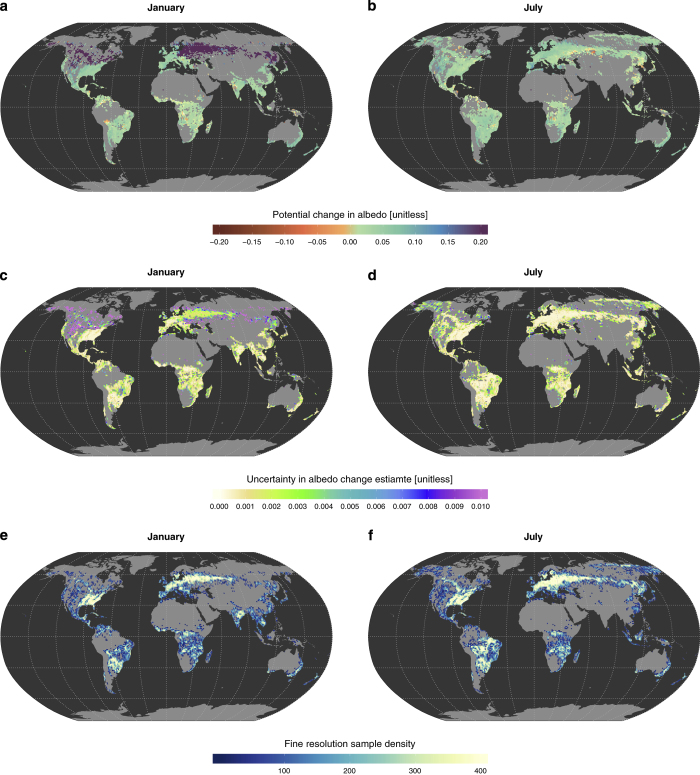
Overview of the information provided for each variable in the dataset. The example presented is albedo for the transition Forest to Crops/Grasses in the broad classification scheme (IGBPgen, see text for details). For the months of January and June: the spatialised variable are shown in **a** and **b**; the uncertainty associated with each value are shown in **c** and **d**; and the number of sample used in the aggregation are shown in **e** and **f**.

Here we present such a dataset, which resulted from a study attempting to characterize the mark of vegetation change on the Earth's surface energy balance^6^. The dataset consists of spatialised information describing the expected changes in surface properties and energy fluxes resulting from specific vegetation cover transitions. The variables provided include the different components of the surface energy balance: shortwave reflected radiation (*SW*_↑_), longwave emitted radiation (*LW*_↑_), latent heat (*LE*) and sensible and ground heat fluxes combined (H+G). The same information is also provided for daytime and nighttime land surface temperature (*LST*_*d*_ and *LST*_*n*_), clear sky longwave emitted radiation (*LW*_↑_*) and albedo (*α*). Two sets of vegetation classes are provided, assuming either 6 broad vegetation transitions or 45 more specific vegetation transitions (e.g. differentiating various forest types). The information is disaggregated at monthly scale for a full seasonal cycle representing the median climatological conditions over the period 2008-2012. All estimates are obtained from satellite measurements from the MODIS instruments at a spatial resolution of 0.05 using a ‘space-for-time’ substitution approach, which are then summarized over 1 grid cells to combine with data from the CERES satellite remote sensing instrument. All values are accompanied by information on uncertainty and the number of samples from which 1 estimates are made, enabling the production of maps such as those shown in Fig. 1.

We anticipate that this dataset can serve to support the development of land-based plans that target climate mitigation. The spatialised estimates could prove to be an important asset for integrated assessment modelling^21^, as well as for benchmarking and improving land-surface schemes and Earth system models^18^. Ultimately, we also expect that our observation-driven dataset could serve as a baseline in the development of monitoring, reporting and verification guidelines for the implementation of land-based biophysical climate mitigation and adaptation options, mirroring what is currently done for biogeochemical land-processes.

## Methods

**Figure 2:**
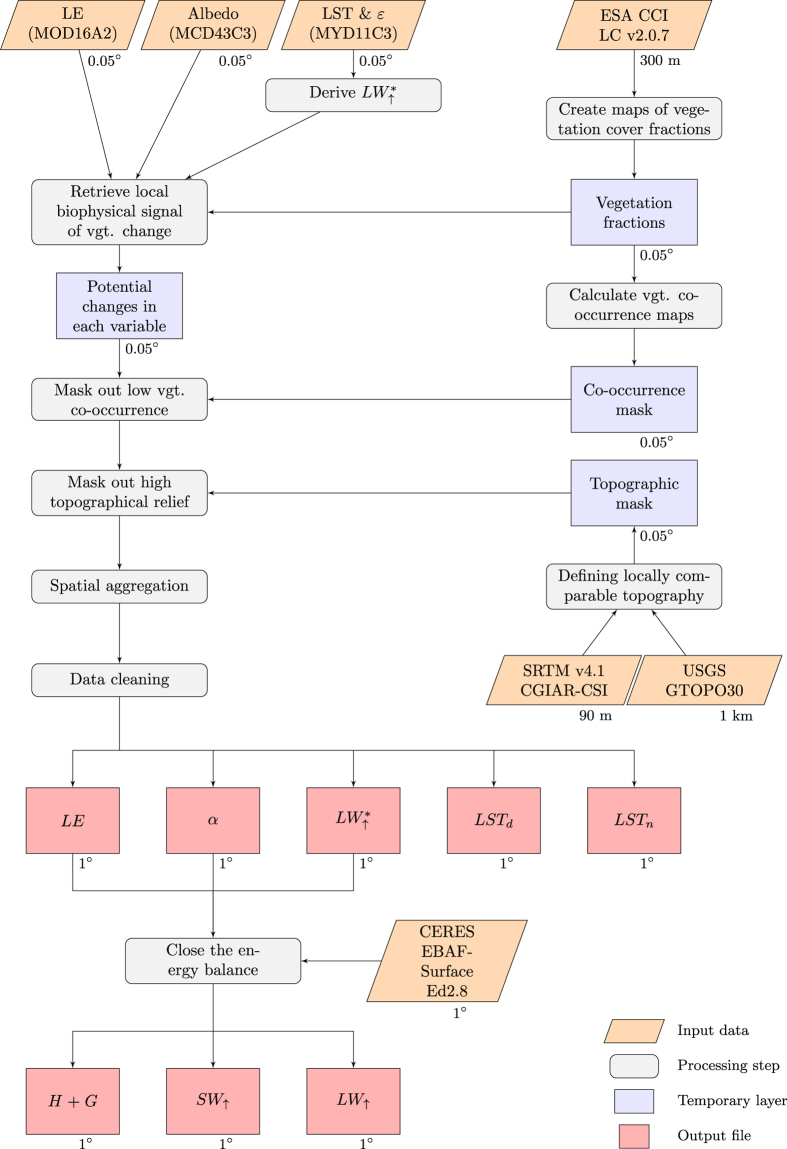
Schematic overview of the processing steps to generate the dataset. The different output files of the dataset correspond to the effects of vegetation cover change for: shortwave reflected radiation (*SW*_↑_), longwave emitted radiation (*LW*_↑_), latent heat (*LE*), sensible and ground heat fluxes combined (*H*+*G*), daytime and nighttime land surface temperature (*LST*_*d*_ and *LST*_*n*_), clear sky longwave emitted radiation (*LW*_↑_*) and albedo (*α*).

The central concept behind the dataset we present here is the combination, over a local moving window, of a static map of vegetation cover fractions with datasets of variables describing surface properties of vegetation that are retrieved from satellite observations. The methodological steps, including steps of masking, aggregating and cleaning, are summarized in the flowchart in Fig. 2. These steps are already described in our related work^6^, but the methodology is also reported here, along with some additional technical details, in order to facilitate the presentation of the dataset.

### Data preparation

The input data for the methodology are of two types: surface property variables that change seasonally and static vegetation cover fraction maps. All have a common spatial resolution of 0.05. The seasonal variables are *α*, *LE*, *LST*_*d*_, *LST*_*n*_ and *LW*_↑_*. They are all derived from measurements from the NASA Moderate Resolution Imaging Spectroradiometer (MODIS) instrument on-board the Aqua and Terra satellites. They are available at monthly temporal resolution, but a median value for each month is calculated from the years 2008 to 2012 to generate the 5-year climatology while retaining the seasonal cycle. The static vegetation cover fraction maps are derived from different satellite instruments and represent a single year: 2010. We describe each variable below, along with specific pre-processing each may have required.

#### Albedo

Albedo (*α*) is defined as the proportion of the incident light or radiation that is reflected by a surface. In this case, we are interested in the monthly average of the proportion of total radiation across the broadband shortwave spectrum reflected by the Earth's surface at 0.05 resolution. The NASA MCD43C3 albedo product provides 8-daily estimates of both directional hemispherical albedo (black-sky albedo) and bihemispherical albedo (white-sky albedo) based on multidate multispectral MODIS cloud-free observations collected over a 16-day moving window and a semi-empirical kernel-driven bidirectional reflectance model^22^. These white-sky and black-sky albedos correspond to theoretical situations in which incident radiation is either completely diffuse or completely direct. To obtain an estimate of real conditions without information on the fraction of diffuse radiation, we took the mean of both values. The mean is preferred here for its simplicity, since other techniques such as weighting or interpolation would require additional information to avoid any subjectivity. To obtain estimates at monthly temporal resolution, we selected those for which the 16-day period best corresponded with the 15th of each month. Data are available from the NASA LPDAAC website (https://lpdaac.usgs.gov/).

#### Latent heat flux

Latent heat flux (*LE*) is the flux of heat from the Earth's surface to the atmosphere that is associated with evaporation of water at the surface. The land component of this flux consists of evaporation of rain water intercepted by the canopy before it reaches the ground, transpiration through stomata on plant leaves and stems, evaporation from wet and moist soil and the sublimation of water vapour from ice and snow; and is otherwise known as terrestrial evapotranspiration (obtained by dividing latent heat flux by the latent heat of vaporization). The MOD16A2 product^23^ provides latent heat obtained by integrating several MODIS products (land cover, albedo, leaf area index, fAPAR) with meteorological data, delivered at 0.05 spatial resolution with monthly temporal resolution covering the regions from 60°S until 80°N. The data are available from the NTSG website (http://www.ntsg.umt.edu/project/modis/mod16.php).

#### Daytime and nighttime LST

These are the radiant temperatures of a surface measured respectively during the day and at night. The MODIS instrument on board the Aqua platform makes such measurements twice over its cycle at approximately 13:30 and 1:30 local time at the Equator. These times are close to those at which the minimum and maximum temperatures are expected. The MODIS land surface temperature algorithm provides such estimates at a monthly time step at 0.05 spatial resolution^24,25^ in the MYD11C3 product (we use collection 5 available from the NASA LPDAAC website https://lpdaac.usgs.gov/).

#### Surface upwelling longwave radiation

This is the outgoing infrared radiation emitted by the surface. A large part of this energy is absorbed by the atmosphere and later re-emitted towards the Earth (by clouds and greenhouse gases) or outwards to space. The upward longwave radiation (*LW*_↑_) can be calculated from the surface temperature (*T*) and broadband emissivity (*ε*_*B*_) using the Stefan-Boltzmann law:

(1)LW↑=εBσT4

where *σ* is the Stefan-Boltzmann's constant (5.67×10^−8^ W m^−2^ K^−4^). We use *LST*_*d*_ and *LST*_*n*_ from the MYD11C3 product to estimate the mean surface temperature (*T*) over the entire day span using a simple average. The MYD11C3 product also provides emissivity estimates for various specific narrow bands in the middle and thermal infrared spectrum that can be used to obtain *ε*_*B*_ using the empirical equation suggested by a dedicated study^26^:

(2)εB=0.2122ε29+0.3859ε31+0.4029ε32

where *ε*_29_, *ε*_31_ and *ε*_32_ are the estimated emissivities in MODIS bands 29 (8400-8700 nm), 31 (10780-11280 nm) and 32 (11770-12270 nm), respectively. Because the satellite can only measure during cloud-free conditions, it must be specified that the resulting monthly upwelling longwave radiation only refers to clear sky conditions, which we will denote using an asterisk: *LW*_↑_*.

#### Fractions of vegetation cover

These are derived from the 300 m global land cover map of the year 2010 provided by the European Space Agency's (ESA) Climate Change Initiative (CCI)^27^. A dedicated tool provided alongside the product enables users to transform the UNLCCS classification scheme as employed^28^ to continuous cover fractions at coarser scales via a ‘crosswalking’ table, typically to characterize the plant functional types used in land surface models^29^. We used this tool to generate two sets of maps for a set of 4 broad classes and another of 10 detailed classes (Table 1) based on the classes of the widely-used global vegetation classification scheme of the International Geosphere Biosphere Programme (IGBP). The crosswalking table we use to pass from UNLCCS to IGBP classifications is provided in Table 2 (available online only).

### Retrieving the local biophysical signal of vegetation change

To identify the biophysical signal due to changes in vegetation cover we establish a relationship between vegetation cover fractions and the surface variables over a local moving window. As a result of this the direct biophysical effects of vegetation change considered here are local. This is valid both for the spatial extent of the cover change, which assumes at most a change of a complete fine resolution pixel (0.05°×0.05°), and for the origin of the change, i.e. we ignore indirect effects due to regional change from neighbouring regions. The moving window size is 5 by 5 pixel at 0.05° resolution, covering an area of approximately 25 km by 25 km over which the local climate is assumed to be uniform. To unmix the signal resulting from the mixed compositional land cover, for each window we apply a linear regression using a matrix ***X*** containing the vegetation fractions of each of the 25 pixels as explanatory variables and a vector *y* containing the 25 values of a given biophysical variable as response variable to obtain a vector of *β* coefficients:

(3)y=Xβ

This is equivalent to solving the following system of equations:

(4){y1=β1x11+β2x12+...+βmx1my2=β1x21+β2x22+...+βmx2m⋮yn=β1xn1+β2xn2+...+βmxnm

in which *x*_*ij*_ represents the cover fraction of vegetation *j* in pixel *i*, for the *n* pixels in the moving window and the *m* classes that are considered. Once identified, we can use the *β* coefficients to predict the local *y* value corresponding to a given composition *x*, including that composed of a single vegetation cover *j* by setting *x*_*j*_=1 and all other *x* values to zero.

There is a problem, however, if the compositional predictor dataset ***X*** is used directly in the analysis. Compositional data can behave somewhat differently to ‘ordinary’, open or normal data, because compositions necessarily sum to one (for this reason they are also sometimes described as ‘closed’ data). Statistically, this can lead to spurious correlations between compositional components, and/or between compositional components and the response variable. Analysis of any given subset of compositional components can lead to very different patterns, results and conclusions^30^. Geometrically, all points defined by the compositions must fall in a simplex because their compositions sum to one. For a three part composition, this simplex is a triangular plane (i.e. it exists on a 2-dimensional surface). Whilst this compositional matrix has 3 columns, there are only (at most) 2 dimensions. A transformation of ***X*** is needed to reduce appropriately the dimensionality of this matrix for subsequent use in the regression.

The transformation we apply to reduce the dimensionality of ***X*** involves a singular value decomposition (SVD). This procedure is very close to a principal component analysis (PCA). The first step consists of centring all the columns of the predictor matrix ***X*** of vegetation fractions by removing the column means. We then apply the SVD:

(5)(X−M)=UDVt

where ***M*** is the appropriate matrix of column means, ***U*** and ***V*** are the matrices containing respectively the lefthand and righthand singular vectors, and ***D*** is a diagonal matrix containing the singular values (the standard deviations of the ensuing dimensions). Squared values of ***D*** indicate how much variance is explained by each (orthogonal) dimension. We implement a rule where as many dimensions from this SVD are retained as to conserve 100% of the original matrix's variation. In doing so, we reduce the dimensionality appropriately as described above, as well as remove what may be additionally redundant dimensions that can occur locally if, for instance, the only points in which 2 classes are represented have exactly the same values. To avoid having problems when there is too little or no information (e.g. if all pixels have exactly the same compositions), we added a pre-condition that there must be at least 10 pixels with different compositions. The final appropriately transformed predictor matrix of reduced dimension ***Z*** is then obtained by:

(6)Z=(X−M)Vz

where the subscript *z* in ***V***_*z*_ indicates that the latter is composed of a subset of righthand singular vectors in ***V*** as selected from ***D*** as described above. The resulting predictor matrix ***Z*** can now be regressed onto the local biophysical variable *y*.

(7)y=Zβ+ε

where ***Z*** has been augmented with a leading column of 1s to accommodate an intercept term in the regression. The standard manner to obtain an estimate of *β* is:

(8)β=(ZtZ)−1Zty

Because the compositional predictor matrix ***X*** has been transformed to matrix ***Z***, regression coefficients identified in the regression of ***Z*** onto *y* do not immediately provide information about the association between the various vegetation cover fractions and the surface property variables. In order to identify the *z* values associated with a particular vegetation (in that local analysis) we instead define a ‘dummy pixel’ whose composition contains only that vegetation class, with all other classes in the dummy pixel's composition set to zero. This pixel's composition is then transformed, and its *y* value predicted. This is the *y* associated with that vegetation type. Since we wish to do this for all compositional components of interest, we actually define a matrix ***P*** with as many rows as these compositional components that we wish to predict. ***P*** is centred on the same column means as above (***M***, specific to each local analysis), and then multiplied by the correct number of transposed right hand singular vectors (***V***_*z*_, again, specific to each local analysis).

(9)Zp=(P−M)Vz

Predicted *y*_*p*_ values for each vegetation type (identified by predicting the appropriately transformed ‘dummy pixels’) are then calculated as:

(10)yp=Zpβ

The expected change in variable *y* associated with a transition from one vegetation type to another at the central pixel of the local window is then the difference between the *y*_*p*_ predicted for each `pure' vegetation type:

(11)ΔyA→B=yB−yA

Beyond our primary interest in the change Δ*y* for a given vegetation transition, we also assess the uncertainty associated with each of these differences. We consider uncertainty in terms of standard deviations, and thus, according to error propagation, the uncertainty for the difference due to the transition from A to B can be determined from:

(12)σA→B=σA2+σB2−2σAB

where *σ*_*A*_^2^ and *σ*_*B*_^2^ are the variances in the estimates of *y* for each vegetation type, and *σ*_*AB*_ is their covariance. This covariance term is important as the uncertainties of the individually predicted *z* values are not independent given that they derive from the same regression model. The variances and covariances of all vegetation types can be obtained from the covariance matrix, which in turn is calculated as:

(13)Σ=ZpVar[β]Zpt

The diagonal terms in Σ are the variances of individual predictions of (individual) vegetation classes. The off-diagonal parts of Σ hold the covariances between these predictions.

The whole procedure described above (variable transformation, regression and uncertainty estimation) is applied globally over 5 by 5 moving windows for the 3 biophysical variables for each of the 12 months of the year at 0.05° spatial resolution for each vegetation transition considered. The regressions are applied to data including information of all vegetation types (either 10 or 4, depending on whether the detailed or broad classification scheme is used) plus 4 non-vegetated classes (urban, water, snow/ice or bare soil), but predictions are only made for vegetated classes. Symmetric transitions yield identical results (e.g. Δ*y*_*A*→*B*_ =− Δ*y*_*B*→*A*_), and thus only a total of (10^2^−10)/2=45 or (4^2^−4)/2=6 transitions are calculated. The resulting maps only provide information for the pixels in which all 25 pixels in the moving window had information.

### Masking out low vegetation co-occurrences

The method relies on there existing co-occurrences of vegetation classes within the local window. Furthermore, the statistical methods that are applied to these sets of points are more likely to provide reliable results when there are large and balanced presences of both vegetation classes of interest. We designed an indicator to quantify how two vectors describing the presences of two classes of vegetation, *p*_*A*_ and *p*_*B*_, represent the potential range of compositional variation at the same time (i.e. how evenly balanced they are against each other, and how abundant they are with respect to the total composition). In a two dimensional space describing the presences of vegetation class A and vegetation class B, we consider a set of *n* points evenly distributed along the line *B*=1−*A*. These points represent the ideal situation and their positions are stored in vectors *q*_*A*_ and *q*_*B*_. For each of these points *q*, we then calculate the Euclidean distance to all points in *p*, and retain only the smallest one that we store in a vector called *d*_*min*_:

(14)dmin=min(qA−pA)2+(qB−pB)2

The situation that would generate the largest cumulated distances is when all *p* points are located in the origin, *i.e.* when there is neither any of class A nor any of class B. This vector of maximum distance *d*_*max*_ is defined as:

(15)dmax=qA2+qB2

The index of vegetation co-occurrence *I*_*c*_ is defined as:

(16)Ic=1−∑idmin,i∑idmax,i

*I*_*c*_ ranges from 0 to 1, corresponding to a gradient of ‘no presence of either class’ to ‘full and evenly balanced presence of both classes’. For each pair of vegetation classes, a threshold of *I*_*c*_<0.5 is used to mask out from the results those pixels whose local windows do not provide enough co-occurrences. This threshold of *I*_*c*_=0.5 represents a situation in which the sum of the minimum distances between all points *p* and *q* is half of the distance between the origin and all *q* points.

### Masking out high topographical variability

Restricting the analysis to a local window reduces the effect of major climatic gradients when comparing biophysical variables of neighbouring pixels. However, climatic gradients can also occur at much finer spatial scales as a result of vertical elevation change in a landscape, or topographical relief. To factor out such a potentially confounding effect, we created a mask to remove areas in which neighbouring pixels should not be compared because of within pixel differences in relief. This mask is constructed based on fine-spatial resolution digital elevation models: the gap-filled SRTM 90 m Digital Elevation Database v4.1^31^ for the region it covers (between 60°S and 60°N) and GTOPO30 at 1 km for the rest of the globe (data available from the U.S. Geological Survey and distributed by the Land Processes Distributed Active Archive Center (LP DAAC), located at USGS/EROS, Sioux Falls, SD. http://lpdaac.usgs.gov). The mean and standard deviation of the elevation is calculated for each 0.05° by 0.05° grid cell to produce two data layers: *μ*_*h*_ and *σ*_*h*_. These two layers are used to derive three indicators of local topographical relief using the values of all *i* pixels in the 5 by 5 pixel moving window. The first, *v*_1_, is just the average standard deviation across the the moving window:

(17)v1=1n∑i=1nΣh,i

High values of *v*_1_ indicate hilly terrain over the considered scale, which should be discarded from the analysis. The second index, *v*_2_, indicates how different the mean elevation within the central pixel is from the average elevation in the local window:

(18)v2=|µh−1n∑i=1nµh,i|

High values of *v*_2_ can further isolate unwanted pixels that may not be identified using *v*_1_ alone. The third, *v*_3_, is similar to *v*_2_ but compares the central pixel's standard deviation of elevation with the standard deviation across the moving window:

(19)v3=|σh−v1|

High values of *v*_3_ can isolate the odd undesirable pixel whose within-pixel elevation has high variance but an average value close to the average height across the local window. These three indicators are combined together in a single layer depicting all pixels satisfying all of the following conditions: *v*_1_<50 m, *v*_2_<100 m and *v*_3_<100 m. Pixels that fail any of these conditions are masked out from all the layers of results.

### Spatial aggregation

The maps resulting from the local space for time analysis need to be spatially aggregated from 0.05° to 1° grid cells to be used alongside data from the CERES instrument, which provides the information necessary to close the energy balance. Aggregating to 1° also has other advantages, namely: (1) a mean difference of a variable associated with change from one vegetation type to another may be assumed to be more accurate than any individual estimate at finer scale; (2) this scale is simpler to map and visualize at global level; and (3) it is more comparable to results from land surface models. Because each 0.05° estimate of Δ*y* includes an associated estimation of its uncertainty, this uncertainty can be used to down-weight less reliable values during the aggregation procedure. The typical approach to do so is weighting based on the inverse of the uncertainty:

(20)Δy-=∑iΔyi/σi2∑i1/σi2

where Δ*y*^®^ is the mean aggregated value, whose uncertainty is calculated as:

(21)σΔy-2=1∑i1/σi2

However, these formulations do not account for the spatial auto-correlation generated by the moving window (1 to 20 pixels may be common between two nearby estimates depending on the possible overlap of their respective 5 by 5 windows). This auto-correlation problem may be compounded further when only a clustered set of 0.05° samples are available within the 1° by 1° area. This can occur due to the topographical masking, or because two vegetation types only co-occur over a small part of the 1° grid cell.

To tackle this auto-correlation, we employ a more generic weighting approach. The weights depend not only on the uncertainties estimated from the regressions as above, but also on how each window is correlated with every other window within the area of 1° grid cell. This information is summarized in a 400 by 400 matrix ***R***_*a*_ containing the fraction of overlap between every pair of windows. The information in ***R***_*a*_ is combined with that of the pixel-wise uncertainties that are embedded in ***D***_*a*_, a diagonal matrix containing the uncertainties in its diagonal, to build a covariance matrix Σ_*a*_ (the subscript *a* is used to differentiate these matrices involved in this aggregation step from those used before):

(22)Σa=DaRaDat

The vector of weights is then obtained as:

(23)w=11tΣa−11Σa−11

which can then be used to calculate the aggregated Δ*ȳ* as:

(24)Δy¯=∑iwiΔyi

while the aggregated uncertainty *σ*_Δ*ȳ*_^2^ is given by:

(25)σΔy¯2=wtΣaw=11tΣa−11

When the windows have no auto-correlations, both equations 24 and 25 fall back to the simpler weighting formulas of equations 20 and 21. The aggregation procedure is applied to all data layers.

### Detection and treatment of outliers

Despite all efforts to characterise uncertainty and reach representative values, the results can still contain unrealistic values. The main reasons for this might be that uncertainties in the input data (the remote sensing surface property variables and the vegetation cover fraction maps) are not explicitly taken into account. As a final step to remove possible outliers, we remove all values for grid cells in which there are not at least 20 samples at 0.05° spatial resolution. Lastly, we also remove values that are statistical outliers based on the distribution of the entire dataset. All data layers are available with their associated uncertainty.

### Closing the surface energy balance

The local unmixing step can only be applied to those variables available at the 0.05° spatial resolution (namely *α*, *LE*, *LW*_↑_*, *LST*_*day*_ and *LST*_*night*_), meaning some components of the surface energy balance are missing. The full surface energy balance is expressed as:

(26)SW↓−SW↑+LW↓−LW↑=H+LE+G

*SW*_↓_, *SW*_↑_, *LW*_↓_ and *LW*_↑_are respectively the downwelling and upwelling radiative fluxes in the shortwave or longwave parts of the spectrum, *LE* is the latent heat flux, *H* is the sensible heat flux and *G* is the ground heat flux. We derive the terms of the energy balance combining MODIS-based datasets with the EBAF-Surface Product derived from the NASA Clouds and the Earth's Radiant Energy System (CERES) instrument. This dataset (CERES EBAF-Surface Ed2.8) provides a closed and gap-filled surface energy balance at 1° that is consistent with CERES top-of-atmosphere irradiance measurements^32^. For the specific goals of this analysis we are interested in how the terms of this equation change according to a change in vegetation cover, i.e.:

(27)ΔSW↓−ΔSW↑+ΔLW↓−ΔLW↑=ΔH+ΔLE+ΔG

We make the assumption that the changes in vegetation cover that are considered here are too small (*i.e.* maximum 0.05°) to generate strong feedbacks in the cloud regime, and as a consequence we assume Δ*SW*_↓_=0 and Δ*LW*_↓_=0. The change in reflected shortwave radiation can be expressed in terms of albedo (*α*) and incoming shortwave radiation (Δ*SW*_↑_=Δ*α*×*SW*_↓_), the latter being available from CERES data at 1°. Although we derived estimates of changes in upwelling longwave flux satellite measurements at 0.05°, these refer to clear-sky conditions only (i.e. when the satellite instrument can measure the ground unobstructed by clouds) while other fluxes are representative of all cloud conditions. As a proxy for the effect of cloudiness, we used a correction factor based on the ratio of all sky (*LW*_*C*↑_) to clear sky (*LW*_*C*↑_*) longwave upwelling estimated by CERES (Δ*LW*_↑_= (Δ*LW*_*C*↑_/*LW*_*C*↑_*)×Δ*LW*_↑_*, where the asterisk indicates values for clear sky conditions). By re-writing and simplifying the equation above, the expression describing the change in the residual flux, composed of both sensible and ground heat fluxes, becomes:

(28)Δ(H+G)=−(Δα)SW↓−(ΔLWC↑/LWC↑)×ΔLW↑−ΔLE

We apply this expression to every 1° pixel for every month of the time-series and every vegetation transition based on the previously calculated datasets of Δ*α*, Δ*LW*_↑_* and Δ*LE*. To have all terms of the energy balance on equal footing and with the same sign convention, we also explicitly produced datasets of shortwave reflected radiation (Δ*SW*_↑_) and full-sky longwave emitted radiation (Δ*LW*_↑_)

### Code availability

Most of the processing has been done using R version 3.3.2. The code can be made available upon request on a case by case basis. The aggregation and transformation of the ESA CCI land cover maps is done using the dedicated user tool (version 3.12) that can be accessed at: http://maps.elie.ucl.ac.be/CCI/viewer/download.php#usertool

### Data Records

The entire dataset (Data Citation 1) is composed of 16 separate netCDF files, as listed in Table 3 (8 variables, for each of two vegetation cover classifications). The 8 variables are: (1) those based only on MODIS data (LE, albedo, LWsfc, LSTday and LSTnight); (2) those requiring a combination of one of the previous along with CERES data (SWreflected and LWemitted); and (3) those requiring a combination of all the former (HG). The vegetation classifications are either detailed (IGBPdet, 10 classes) or generic (IGBPgen, 4 classes) as described in the methods section and in table 1. The names of the netCDF files include both the variable and the land cover classification scheme (e.g. HG_IGBPdet.nc for Δ(*H*+*G*) given the detailed land cover scheme, and that therefore includes 45 transitions).

All files provide information across the global domain at a spatial resolution of 1°×1° and monthly temporal resolution over a climatological year representing the period 2008-2012. Each netCDF file has the same structure based on 4 different dimensions: latitude (lat), longitude (lon), month (mon) and vegetation transition code (iTr). Three dependent variables are recorded over these 4 dimensions for each variable *Z*: the difference in variable *Z* for a given vegetation transition (Delta_Z), the uncertainty (provided as a standard deviation) associated with the estimation of this difference in *Z* (SD_Delta_Z), and the number of fine spatial resolution (0.05°×0.05°) samples from which each aggregated (1°×1°) value is derived (N_Z).

The vegetation transition code (iTr) is a two or three digit number, representing the class of origin (first digit), and the class of destination (second, or second and third digit), with classes coded as in table 1. For example, for the 10 class system IGBPdet, the code iTr=19 represents the transition from class 1 (EBF) to class 9 (CRO), while the code iTr=23 represents the transition from class 2 (DBF) to class 3 (ENF). Because there are 10 classes, any transition from classes 1-9 to class 10 will result in a three digit transition code (e.g. iTr=110 represents the transition from class 1 (EBF) to class 10 (wetlands, WET)). There is no ambiguity with a three digit transition code, they are always from a (1 digit) class to class 10. Transitions from a larger number to a smaller number are not encoded as they are equal to the transition from the smaller number to the greater number, albeit with a change of sign (e.g. iTr=32 would be equivalent to iTr=23 with a negative sign added to the mean change).

### Technical Validation

**Figure 3:**
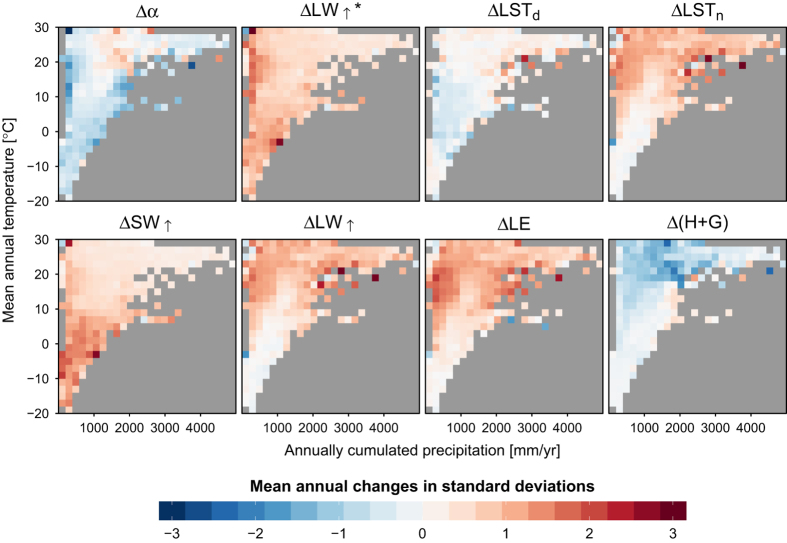
Summary of annual changes in all variables due to a given type of vegetation cover change plotted in a climatic space. Values of a given biophysical variable have been divided by the standard deviation of the entire population prior to aggregation in climate space. The climate values come from CRUNCEP and cover the period 2008–2012.

The dataset successfully reproduces the major patterns that are expected following vegetation cover change. Figure 3 reports the mean annual effect of deforestation for each of the 8 variables in the dataset, summarized across climatic gradients of mean annual temperature and annually cumulated precipitation (both from CRU data v4.00 at 0.5°× 0.5° resolution). The change in albedo (and shortwave reflected radiation) is a consistent brightening in all climates (since forest are generally darker than crops or grasses^1^), but with much higher values in cold climates, where the snow effect is expected to exacerbate the change, and very dry areas, where grasses remain dry for large parts of the year or crops are not so densely sown and crop seasons are shorter, allowing a stronger influence of the bright dry soil on the signal. The change in longwave emitted radiation and daytime LST is stronger in warmer climates where an increase is expected caused by the strong decrease in latent heat, which in turn is caused by the fact that herbaceous plants typically have shallower roots and less access to water than trees. The changes in nighttime LST are milder than those during daytime, but they do show a change in sign, with a decrease in colder climates and a slight increase in warmer ones. The reason behind this is probably the reduction in surface roughness going from trees (particularly needleleaf which dominate in colder climates) to crops/grasses, causing a stable stratification of the air at night, which is particularly evident in colder climates and at lower levels of radiation. The changes in the residual fluxes (*H*+*G*) are the consequence of all the other fluxes in the surface energy balance.

**Figure 4:**
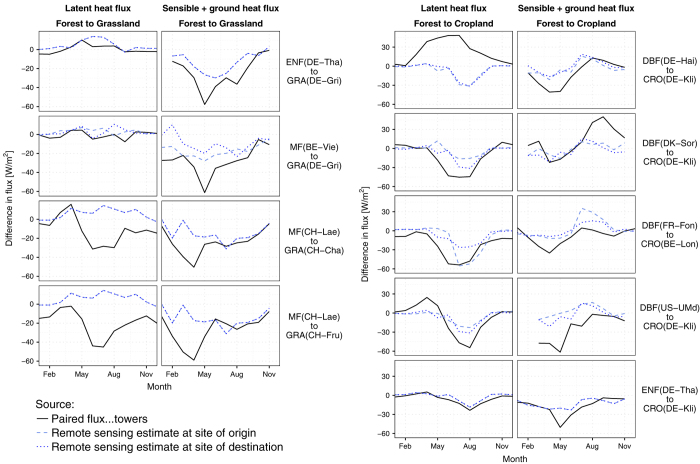
Comparison of the dataset against individual paired sites of flux-tower measurements.

**Figure 5:**
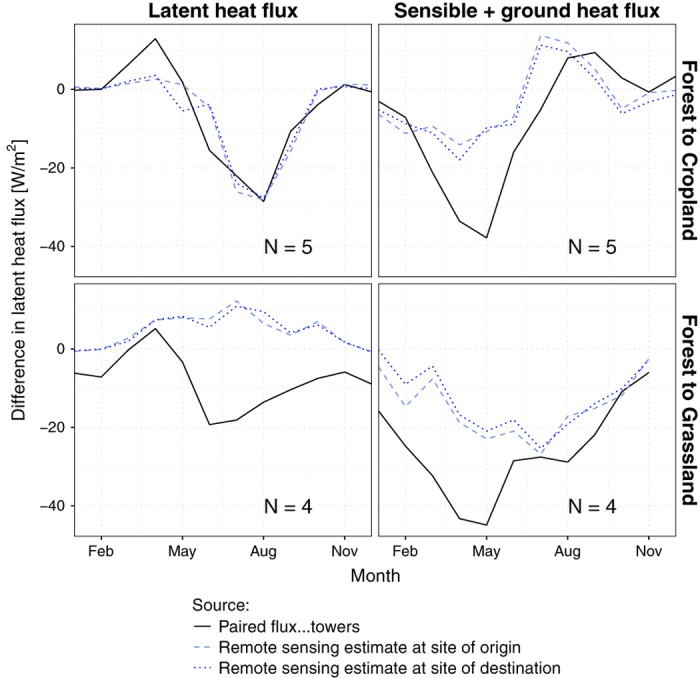
Comparison of the dataset against averaged paired sites of flux-tower measurements. The value of N indicates the number of paired sites involved in the average.

A validation with ground-based measurements of surface energy balance fluxes would be desirable to make a proper detailed evaluation of the present dataset. However, no such network of measurements currently exists. The closest candidate are the flux-tower measurements from FLUXNET2015, but there are insufficient sites with comparable climate conditions and contrasting vegetation types to cover the spatial and thematic (i.e. vegetation type transitions) extents in the datasets. In the absence of proper validation, an indicative evaluation is proposed based on a selection of paired FLUXNET sites that satisfy similarity criteria based on ERA-Interim reanalysis data at each site. These criteria require that to be paired, sites must have: (1) less than 10% difference in cumulated annual precipitation; (2) less than 2°C difference in mean annual air temperature; and (3) similar degree of continentality, defined as less than 0.5° difference in standard deviation of monthly air temperature. Only paired sites including transitions between forests and grasses or crops are considered, resulting in 9 pairs whose difference in turbulent fluxes are compared with the values from the present dataset at each site in Fig. 4 (radiative fluxes were not sufficiently available in the dataset). These comparisons are not expected to match closely as the spatial supports are radically different: the flux-tower measurements represent a specific patch of area of less than 1 km^2^ while the remote sensing data pixels cover the average conditions over 1°×1°. However, the seasonal profiles of our datasets do generally show a reasonable degree of similarity with those from the paired sites, and this similarity increases when they are aggregated according to general classes as shown Fig. 5.

### Usage Notes

Several assumptions were necessary when constructing this dataset, and should be considered when interpreting or reusing it. An important point to emphasise is that, although the data layers are provided with a spatial resolution of 1°, the values themselves represent a spatial average of effects that would occur for vegetation cover change at the scale of 0.05° or less, and thus represent direct local effects. A total change of vegetation cover over an area of 1°×1°, which is highly unrealisitic, would potentially cause a much larger perturbation involving both direct and indirect effects.

We also assume vegetation cover is the only driver of changes in biophysics within the local moving window of 0.25°. Areas with strong elevation gradients are masked out to filter topographic effects, but other changes, in soil properties for example, are disregarded. To close locally the surface energy balance, we also need to assume that changes of vegetation cover within the 0.05°×0.05° area do not generate indirect cloud feedbacks with the atmosphere that would further change the energy balance. For example, due to their higher roughness forested landscapes can induce more cloud formation than grasslands^33^, and these small clouds can change the energy balance by reducing incoming shortwave radiation, increasing incoming longwave radiation and exporting latent heat. Differences in soil moisture under contrasting vegetation covers could also contribute to indirect changes in the surface energy balance that cannot be resolved in this dataset. Assessing these kind of effects would require the use of land surface models coupled with dynamic regional climate models which are beyond the scope of this work. The assumption to neglect these feedbacks relies on the fine scale of the analysis and on the typical lateral movement of air masses due to wind that ultimately advect air masses to different grid cells.

The dataset is based on satellite observations obtained during the period 2008–2012. These values should remain valid for various years in the past or the future if the background climate does not change substantially^9^. Changes in background climate, such as an average increase in temperature that reduces the length of the snow cover period or intensifies heat stress in summer, will change the albedo and the evapotranspiration signal respectively. User are not encouraged to project these values in scenarios of future climates without considering carefully the associated changes in climate at every pixel.

## Additional information

**How to cite this article:** Duveiller G. *et al.* A dataset mapping the potential biophysical effects of vegetation cover change. *Sci. Data* 5:180014 doi: 10.1038/sdata.2014.14 (2018).

**Publisher’s note:** Springer Nature remains neutral with regard to jurisdictional claims in published maps and institutional affiliations.

## Supplementary Material



## Figures and Tables

**Table 1:** Classes of vegetation cover used in the dataset.

**Classification scheme**	**Vegetation class full name**	**Abbreviation**	**Code**
IGBPdet	Evergreen Broadleaf Forest	EBF	1
IGBPdet	Deciduous Broadleaf Forest	DBF	2
IGBPdet	Evergreen Needleleaf Forest	ENF	3
IGBPdet	Deciduous Needleleaf Forest	DNF	4
IGBPdet	Mixed Forest	MF	5
IGBPdet	Savannas	SAV	6
IGBPdet	Shrublands	SHR	7
IGBPdet	Grasslands	GRA	8
IGBPdet	Cropland	CRO	9
IGBPdet	Wetlands	WET	10
IGBPgen	Forests	FOR	1
IGBPgen	Shrublands	SHR	2
IGBPgen	Crops & grasses	C+G	3
IGBPgen	Savannas	SAV	4
The codes indicate the numbers used to identify the classes in the data layers for both detailed IGBPdet and IGBPgen broad classifications schemes.			

**Table 2:** Cross-walking table used to translate land cover classes of the FAO LCCS system used in the ESA CCI maps to IGBP classes.

	**LCCS Class IGBP**	**IGBPgen:**	**FOR**	**FOR**	**FOR**	**FOR**	**FOR**	**SAV**	**SHR**	**C+G**	**C+G**	**WET**	**URB**	**WAT**	**SNO**	**BSV**
		**IGBPdet:**	**EBF**	**DBF**	**ENF**	**DNF**	**MF**	**SAV**	**SHR**	**GRA**	**CRO**	**WET**	**URB**	**WAT**	**SNO**	**BSV**
**10**	Cropland, rainfed									100						
**11**	Herbaceous cover										100					
**12**	Tree or shrub cover										100					
**20**	Cropland, irrigated or post-flooding										100					
**30**	Mosaic cropland (>50 %) nat. veg. (tree, shrub, herb.) (<50 %)										100					
**40**	Mosaic nat. veg. (tree, shrub, herb.) (>50 %)/cropland (<50 %)										100					
**50**	Tree cover, broadleaf, evergreen, closed to open (>15 %)		100													
**60**	Tree cover, broadleaf, deciduous, closed to open (>15 %)			100												
**61**	Tree cover, broadleaf, deciduous, closed (>40 %)			100												
**62**	Tree cover, broadleaf, deciduous, open (15–40 %)							100								
**70**	Tree cover, needleleaf, evergreen, closed to open (>15 %)				100											
**71**	Tree cover, needleleaf, evergreen, closed (>40 %				100											
**72**	Tree cover, needleleaf, evergreen, open (15–40 %)							100								
**80**	Tree cover, needleleaf, deciduous, closed to open (>15 %)					100										
**81**	Tree cover, needleleaf, deciduous, closed (>40 %)					100										
**82**	Tree cover, needleleaf, deciduous, open (15–40 %)							100								
**90**	Tree cover, mixed leaf type (broadleaf and needleleaf						100									
**100**	Mosaic tree and shrub (>50 %)/herbaceous cover (<50 %)							100								
**110**	Mosaic herbaceous cover (>50 %)/tree and shrub (<50 %)							100								
**120**	Shrubland								100							
**121**	Shrubland evergreen								100							
**122**	Shrubland deciduous								100							
**130**	Grassland										100					
**140**	Lichens and mosses															100
**150**	Sparse vegetation (tree, shrub, herbaceous cover) (<15 %)															100
**152**	Sparse shrub (<15 %)															100
**153**	Sparse herbaceous cover (<15 %)															100
**160**	Tree cover, flooded, fresh or brackish water											100				
**170**	Tree cover, flooded, saline water											100				
**180**	Shrub/herbaceous cover, flooded, fresh/saline/brackish water											100				
**190**	Urban areas												100			
**200**	Bare areas															100
**201**	Consolidated bare areas															100
**202**	Unconsolidated bare areas															100
**210**	Water bodies													100		
**220**	Permanent snow and ice														100	
The values indicate the percent contribution of each LCCS class, which normally can vary, but have here been all set to 100%.																

**Table 3:** List of separate files contained in the dataset.

**Variable**	**Classification**	**Source**	**Method**	**Data**
Albedo	IGBPdet	MODIS (MCD43C3)	Direct retrieval	albedo_IGBPdet.nc
Latent heat	IGBPdet	MODIS (MOD16A2)	Direct retrieval	LE_IGBPdet.nc
Longwave outgoing (clear sky)	IGBPdet	MODIS (MYD11C3)	Direct retrieval	LWsfc_IGBPdet.nc
Daytime land surface temperature	IGBPdet	MODIS (MYD11C3)	Direct retrieval	LSTday_IGBPdet.nc
Nighttime land surface temperature	IGBPdet	MODIS (MYD11C3)	Direct retrieval	LSTnight_IGBPdet.nc
Shortwave reflected	IGBPdet	CERES EBAF Surface (Ed2.8), Albedo	Combination with CERES	SWreflected_IGBPdet.nc
Longwave emitted (full sky)	IGBPdet	CERES EBAF Surface (Ed2.8), Longwave outgoing (clear sky)	Combination with CERES	LWemitted_IGBPdet.nc
Combined sensible and ground heat fluxes	IGBPdet	Shortwave reflected, Latent heat, Longwave outgoing (clear sky)	Energy balance closure	HG_IGBPdet.nc
Albedo	IGBPgen	MODIS (MCD43C3)	Direct retrieval	albedo_IGBPgen.nc
Latent heat	IGBPgen	MODIS (MOD16A2)	Direct retrieval	LE_IGBPgen.nc
Longwave outgoing (clear sky)	IGBPgen	MODIS (MYD11C3)	Direct retrieval	LWsfc_IGBPgen.nc
Daytime land surface temperature	IGBPgen	MODIS (MYD11C3)	Direct retrieval	LSTday_IGBPgen.nc
Nighttime land surface temperature	IGBPgen	MODIS (MYD11C3)	Direct retrieval	LSTnight_IGBPgen.nc
Shortwave reflected	IGBPgen	CERES EBAF Surface (Ed2.8), Albedo	Combination with CERES	SWreflected_IGBPgen.nc
Longwave emitted (full sky)	IGBPgen	CERES EBAF Surface (Ed2.8), Longwave outgoing (clear sky)	Combination with CERES	LWemitted_IGBPgen.nc
Combined sensible and ground heat fluxes	IGBPgen	Shortwave reflected, Latent heat, Longwave outgoing (clear sky)	Energy balance closure	HG_IGBPgen.nc
