# Addendum: A dataset mapping the potential biophysical effects of vegetation cover change

**DOI:** 10.1038/s41597-020-00787-6

**Published:** 2021-01-11

**Authors:** Gregory Duveiller, Josh Hooker, Alessandro Cescatti

**Affiliations:** European Commission Joint Research Centre, Directorate D-Sustainable Resources-Bio-Economy Unit, I -21027, Ispra, (VA) Italy

Addendum to: *Scientific Data* 10.1038/sdata.2018.14, published online 20 February 2018

The original dataset^1^ provides spatialised estimates of how a series of biophysical environmental variables (e.g. daytime land surface temperature, latent heat, etc.) would locally change following potential changes in vegetation cover. The data-driven methodology imposed a limitation on the spatial extent over which these estimates could be made, as it required both source (what is transitioned from) and target (what is transitioned to) vegetation cover types to co-exist within a local moving window. To provide a more widespread spatial coverage as required by some users, this second version of the dataset has been expanded to places where only one of the two vegetation classes involved in a given transition occurs. This is achieved using Random Forests^2^, a non-linear regression tree approach, to predict the change in biophysical variables based on local climate. The method employed is described in (Duveiller *et al*. 2020)^3^, but is here applied using the updated version ts4.04 of the CRU climate dataset^4^ and an extra climatic predictor describing snow presence (as the cumulated precipitation during days with negative temperatures in degree Celsius). The other predictors are mean temperature, temperature range, cumulated precipitation and an aridity index.

Both the original and updated datasets are now hosted in the JRC Data Catalogue^5^.

More specifically, the new version (v2.0) of the dataset is available at: https://jeodpp.jrc.ec.europa.eu/ftp/jrc-opendata/ECOCLIM/Biophysical-effects-vgt-change/v2.0/ this new version supersedes the previous version (v1.0), which can still be found either at either: https://jeodpp.jrc.ec.europa.eu/ftp/jrc-opendata/ECOCLIM/Biophysical-effects-vgt-change/v1.0/; or at: figshare^6^.

Code necessary to transform v1.0 to v2.0 is available at Zenodo^7^.

The updated data have the same format as before, but now contain an extra variable with an *_ext* suffix, which is the spatially extended version. The original data (without the *_ext* suffix) are also available in each file. Note that over the same location both will differ as the extended version is in effect performing a smoothing operation due to the climate-based interpolation. Finally, the file sizes have been substantially reduced by using the internal compression within the netCDF files.
